# Single nucleotide polymorphisms reveal genetic diversity in New Mexican chile peppers (*Capsicum* spp.)

**DOI:** 10.1186/s12864-021-07662-7

**Published:** 2021-05-17

**Authors:** Dennis N. Lozada, Madhav Bhatta, Danise Coon, Paul W. Bosland

**Affiliations:** 1 Department of Plant and Environmental Sciences, New Mexico State University, NM 88003 Las Cruces, USA; 2 Chile Pepper Institute, New Mexico State University, 88003 Las Cruces, NM USA; 3 Bayer Crop Science, 63017 Chesterfield, MO USA

**Keywords:** *Capsicum* spp., Chile peppers, Genetic diversity, Genotyping-by-Sequencing, Linkage disequilibrium, Population structure, Single nucleotide polymorphism markers

## Abstract

**Background:**

Chile peppers (*Capsicum* spp.) are among the most important horticultural crops in the world due to their number of uses. They are considered a major cultural and economic crop in the state of New Mexico in the United States. Evaluating genetic diversity in current New Mexican germplasm would facilitate genetic improvement for different traits. This study assessed genetic diversity, population structure, and linkage disequilibrium (LD) among 165 chile pepper genotypes using single nucleotide polymorphism (SNP) markers derived from genotyping-by-sequencing (GBS).

**Results:**

A GBS approach identified 66,750 high-quality SNP markers with known map positions distributed across the 12 chromosomes of *Capsicum*. Principal components analysis revealed four distinct clusters based on species. Neighbor-joining phylogenetic analysis among New Mexico State University (NMSU) chile pepper cultivars showed two main clusters, where the *C. annuum* genotypes grouped together based on fruit or pod type. A Bayesian clustering approach for the *Capsicum* population inferred *K* = 2 as the optimal number of clusters, where the *C. chinense* and *C. frutescens* grouped in a single cluster. Analysis of molecular variance revealed majority of variation to be between the *Capsicum* species (76.08 %). Extensive LD decay (~ 5.59 Mb) across the whole *Capsicum* population was observed, demonstrating that a lower number of markers would be required for implementing genome wide association studies for different traits in New Mexican type chile peppers. Tajima’s D values demonstrated positive selection, population bottleneck, and balancing selection for the New Mexico *Capsicum* population. Genetic diversity for the New Mexican chile peppers was relatively low, indicating the need to introduce new alleles in the breeding program to broaden the genetic base of current germplasm.

**Conclusions:**

Genetic diversity among New Mexican chile peppers was evaluated using GBS-derived SNP markers and genetic relatedness on the species level was observed. Introducing novel alleles from other breeding programs or from wild species could help increase diversity in current germplasm. We present valuable information for future association mapping and genomic selection for different traits for New Mexican chile peppers for genetic improvement through marker-assisted breeding.

**Supplementary Information:**

The online version contains supplementary material available at 10.1186/s12864-021-07662-7.

## Introduction

Chile peppers belonging to the genus *Capsicum* are one of the most important vegetable crops in the world. Domestication of *Capsicum* is believed to have started thousands of years ago in Mexico or North Central America. Previous analyses dated wild chile harvesting from ~ 8,000 years ago, followed by the cultivation and domestication of the *C. annuum* ~ 6,000 years ago [1, 2]. Another study based on species distribution modeling and paleobiolinguistics combined with genetic and archaeobotanical data confirmed that chile pepper domestication originated in central-east Mexico [3]. At present, there are five known domesticated species, namely *C. annuum* L., *C. baccatum* L., *C. chinense* Jacq., *C. frutescens* L., and *C. pubescens* Ruiz & Pav., [3] with many important applications in health, culinary, agriculture, and industry [4, 5].

With new genotyping platforms and techniques being developed, it would be relevant to perform more comprehensive genotyping and sampling with enhanced genomic coverage to better understand diversification under domestication [6]. Next-generation sequencing (NGS) approaches have revealed the rich, dynamic genetic architecture of the chile pepper genome. De novo genome sequencing of “Criollo de Morellos 334” (CM-334), a Mexican landrace that consistently shows resistance to a variety of pathogens including *Phytophthora capsici*, for instance, demonstrated that heat level started through the evolution of new genes by the unequal duplication of existing genes and changes in gene expression following speciation [7]. Whole-genome resequencing of cultivated and wild chile peppers further revealed that the chile pepper genome has expanded ~ 0.30 million years ago through a rapid amplification of retrotransposons consequently resulting in more than 80 % repetitive sequences [8]. More recently, the role of transposable elements on the formation of new genome structure in *Capsicum* has been demonstrated, and the key roles of retroduplication in the emergence of major disease-resistance genes in chile peppers has been revealed [9]. By examining the whole landscape of the chile pepper genome, insights into the genes, gene products, and genetic pathways related to important traits in *Capsicum* will be expanded.

The availability of whole genome sequences for chile pepper [7, 9] allows for the effective implementation of a genotyping by sequencing (GBS) approach for genotyping and genome wide marker discovery of single nucleotide polymorphisms (SNP) for assessment of genetic relatedness among breeding populations. Due to their abundance in the genome, flexibility, speed, cost-effectiveness, and ease of genetic data management, SNPs have become a marker of choice in plant breeding [10, 11]. As an NGS system, GBS has been developed as a fast and robust genotyping method for reduced-representation sequencing of multiplexed samples for genotyping and molecular marker discovery and is a superior platform for plant breeding applications [12, 13]. A GBS approach includes genomic DNA digestion with restriction enzymes to reduce genome complexity, followed by ligation of barcode adapters, PCR, and sequencing of the amplified DNA [14, 15]. Due to its cost-effectiveness and versatility, GBS has been applied for genomics-assisted breeding of important traits on several crops such as rice (*Oryza sativa*) [16], wheat (*Triticum aestivum*) [17], soybean (*Glycine max*) [18], tomato (*Solanum lycopersicum*) [19], and eggplant (*S. melongena*) [20], among others. In chile peppers, GBS-derived SNP markers have characterized genetic diversity, genetic stratification, and relatedness among a collection of Spanish landraces, where population structure was related with fruit morphology and geographic origin [21]. Similarly, a collection of 222 *C. annuum* cultivars characterized using high-density SNP showed clustering not only on geographical origin, but also based on fruit-related traits [22]. In another study, Taitano et al. [6] evaluated a Mexican chile pepper collection using SNP markers and observed that genetic diversity was related to the cultivation techniques used for the different landraces.

Genetic diversity, which represents the magnitude of genetic variability within a population, is an important source of biodiversity [23] and is relevant for association studies, genomic selection, and individual identification, and is crucial to the overall success of plant breeding programs [24, 25]. Diversity in plant genetic resources provides avenues for plant breeders to develop novel cultivars with improved characteristics such as yield potential, pest and disease resistance, and productivity [26, 27]. Genetic diversity studies are important for the genetic fingerprinting of varietal types, identification of genetic relatedness among different genotypes for breeding programs, genetic resource conservation, and development of non-redundant core collections [21].

Chile peppers are among the major crops in the State of New Mexico, with the official state question, “*Red or Green*?” referring to these valuable crops. Genetic diversity analysis of New Mexican chile peppers using high-density genome wide markers, however, remains lacking and therefore it would be relevant to evaluate diversity for breeding and development of improved pepper cultivars for farmers and consumers. The current study used GBS-derived SNP markers to assess the level of genetic diversity, linkage disequilibrium, and population structure among New Mexican chile peppers. DNA profiling could identify beneficial alleles and their combinations that could be introduced in different chile pepper breeding programs for the genetic improvement of current germplasm. Information from this study will be a valuable resource for future association mapping and genomic selection for important horticultural traits in chile peppers.

## Results

### Genotyping-by-sequencing derived SNP markers

Sequencing using Illumina NovaSeq™ 6000 generated an average of 4.31 million high-quality read tags for the 165 chile pepper genotypes. After further processing and quality control based on various filtering criteria, 75,839 SNP markers distributed across the 12 chromosomes of *Capsicum* were discovered. Out of this number, 66,750 SNP markers (88 %) (Additional file 1, Table S1**;** www.10.6084/m9.figshare.14447526) have known map positions in the *Zunla-1* reference genome [8]. Only the markers with known positions were used for genetic diversity analysis. Average frequency of minor allele for the 66,750 SNP loci was 0.21, and the proportion of heterozygotes was 0.05. Across the SNP sites, the most common allele was the ‘G’ allele (23.84 %), followed by ‘A’ (23.79 %), ‘T’ (23.55 %), and ‘C’ (23.52 %). Altogether, 5.31 % of the sites have ambiguous nucleotide calls. Chromosomes P3 (9,250 SNP markers), P1 (7,365), and P2 (6,987) had the highest number of markers, whereas P11 (3,915), P9 (4,024), and P5 (3,915) had the least number of SNP loci. In total, 38,587 (57.80 %) of the SNP sites have transition substitutions, whereas 28,163 (42.20 %) have transversions.

### Analysis of molecular variance and principal components

**Fig. 1:**
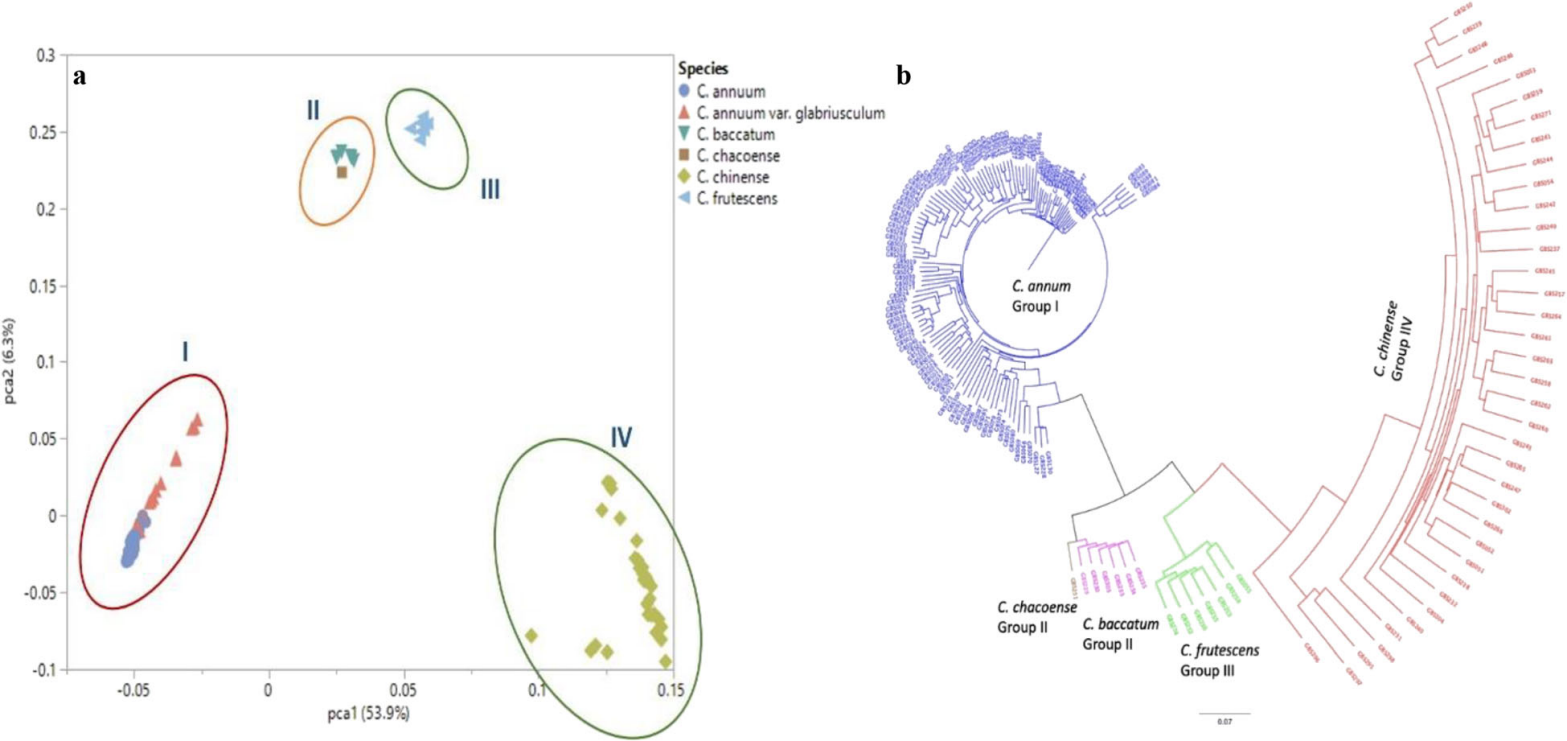
**a** Principal component (PC) biplot derived from genome wide SNP marker data for the *Capsicum* population showing four major clusters based on species. Group I comprised of the *C. annuum* and *C. annuum* var. *glabriusculum* (chiltepins); Group II consisted of *C. baccatum* and *C. chacoense*; and Groups III and IV comprised of *C. frutescens* and *C. chinense*, respectively. **b** Neighbor-joining tree for the *Capsicum* population showing differentiation based on species. *C. annuum* (Group I), *C. frutescens* (Group III) and *C. chinense* (Group IV) formed distinct clusters, whereas *C. baccatum* and *C. chacoense* formed a separate group (Group II), similar with what was observed in the PC plot

Analysis of molecular variance using genome wide SNP markers revealed majority of variation to be among the *Capsicum* populations (76.08 %) (Table 1). Variations among samples within a population accounted for 14.28 %, whereas within sample variation was 9.64 %. Principal components analysis (PCA) revealed four major groups based on species (Fig. 1a). The *C. annuum* and the chiltepins (*C. annuum* var. *glabriusculum*; considered as the progenitors of domesticated *C. annuum* var. *annuum*) formed a distinct cluster (Group I), whereas *C. baccatum* and *C. chacoense* formed the second group. The *C. frutescens* and *C. chinense* represented Groups III and IV, respectively. The first principal component (PC1) accounted for 53.9 % of variation, whereas PC2 accounted for 6.3 % of the total variation.

**Fig. 2:**
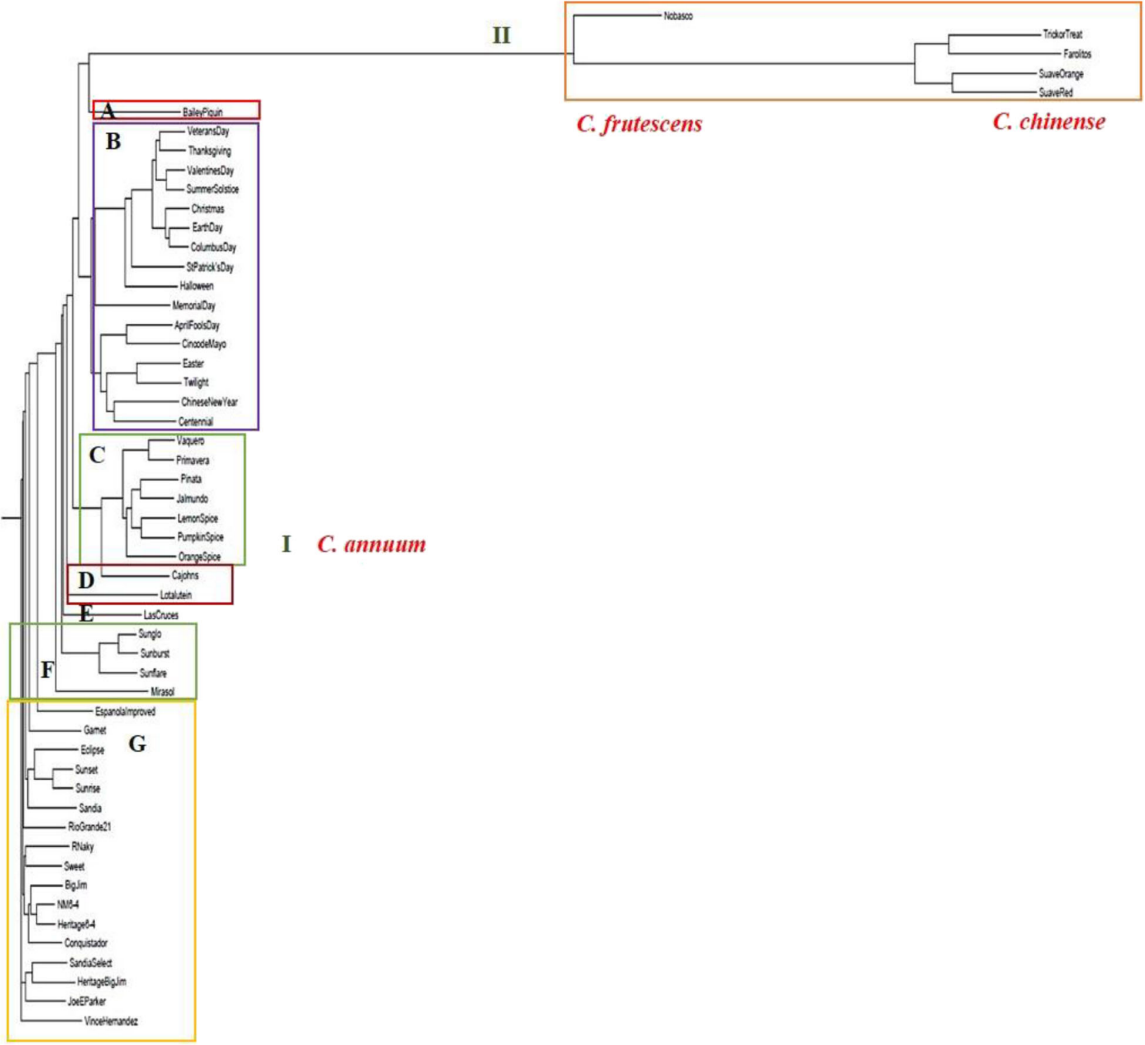
Neighbor joining (NJ) phylogenetic tree for the NMSU (‘NuMex’) chile pepper cultivars based on genome wide SNP markers. Cultivars were divided into two major clusters (I and II) according to species. The *C. annuum* (Cluster I) was separated into seven subgroups (**a**-**g**) based on pod (fruit) types: **a** chile piquin; **b** ornamental chile peppers; **c** jalapeno; **d** serrano; **e** cayenne; **f** de arbol; and **g** New Mexican (includes the paprika type). *C. frutescens* and *C. chinense* formed Cluster II that comprised of the tabasco and the habanero types, respectively. Note that the official names for the NMSU chile pepper cultivars include the designation ‘NuMex’ before the actual name, e.g. ‘Numex Nobasco’. For convenience, the name was omitted in the NJ tree presented herein

Results from the PCA were consistent with clustering based on a neighbor-joining (NJ) phylogenetic analysis for the *Capsicum* population (Fig. 1b). A NJ genetic analysis for NMSU chile pepper cultivars revealed two distinct clusters based on species (Fig. 2). The *C. annuum* cultivars formed a separate group, whereas *C. frutescens* and *C. chinense* clustered together. Within the NMSU *C. annuum* group (Cluster I), there were seven subclusters differentiated based on their fruit or pod type. Group A consisted of the chile piquin, whereas the ornamental chile peppers comprised Group B. The jalapeno types comprised Group C, and Group D contained the serrano peppers. Groups E and F consisted of the cayenne and de arbol types, respectively. Finally, Group G comprised of the New Mexican chile peppers, including the paprika type. Cluster II (*C. frutesce*ns and *C*. *chinense*) comprised of the tabasco and habanero types, respectively, on separate branches.

**Table 1:** Analysis of molecular variance using genome wide SNP markers for the *Capsicum* populations

	Df^a^	SS	MS	σ	%
Between population	3	2181446.0	727148.7	13965.98	76.08
Between samples within population	161	1128947.0	7012.09	2621.46	14.28
Within samples	165	291914.6.0	1769.18	1769.18	9.64
Total	329	3602308.0	10949.30	18356.60	100

^a^*Df * Degrees of freedom; *SS *Sum of Squares; *MS *Mean Square

### Genetic diversity

Various measures of genetic diversity are presented in Table 2. The level of observed heterozygosity (*H*_*o*_) across the population was 0.06. Both the *C. annuum* (Group I) and *C. baccatum* and *C. chacoense* (Group II) complexes had an *H*_*o*_ of 0.04. *C. frutescens* (Group III) and *C. chinense* (Group IV) had *H*_*o*_ values of 0.05 and 0.10, respectively. Inbreeding coefficient for the *Capsicum* population was 0.54. Within the groups, Group I (*C. annuum*) had the highest coefficient of inbreeding (0.70), followed by Group IV (*C. chinense*) (0.51). Group II (*C. baccatum* and *C. chacaoense*) had the least value for inbreeding coefficient (0.34). Gene diversity (*H*_*s*_) was highest among the *C. chinense* (0.20), followed by the *C. annuum* (0.13), and *C. frutescens* (0.08). The whole *Capsicum* population had an *H*_*s*_ value of 0.12. Observed nucleotide diversity (π) across the whole population was 0.33. Within the species, *C. chinense* had the highest π (0.17), followed by the *C. annuum* var. annuum and *C. annuum* var. *glabriusculum* complex (0.12). Expected nucleotide diversity (θ) for the whole *Capsicum* panel was 0.18. Similarly, within the individual species, *C. chinense* had the highest value for θ, followed by the *C. annuum* and chiltepin complex with 0.19 and 0.13, respectively. Fixation index (*F*_*st*_) among the different *Capsicum* species was highest for *C. annuum* and *C. chinense* (0.71), followed by *C. annuum* and *C. frutescens* (0.61) and *C. annuum* and *C. baccatum* and *C.chacoense* complex (0.55) (Additional file 2, Table S1). *C. frutescens* and *C. baccatum* and *C. chacoense* had an *F*_*st*_ value of 0.38, whereas *C. chinense* and *C. baccatum and chacoense* complex had an *F*_*st*_ of 0.48. Polymorphism information content (PIC) values ranged between 0.02 (*C. baccatum* and *C. chacoense*) and 0.12 (*C. chinense*). The PIC value across the whole *Capsicum* population was 0.30.

**Fig. 3:**
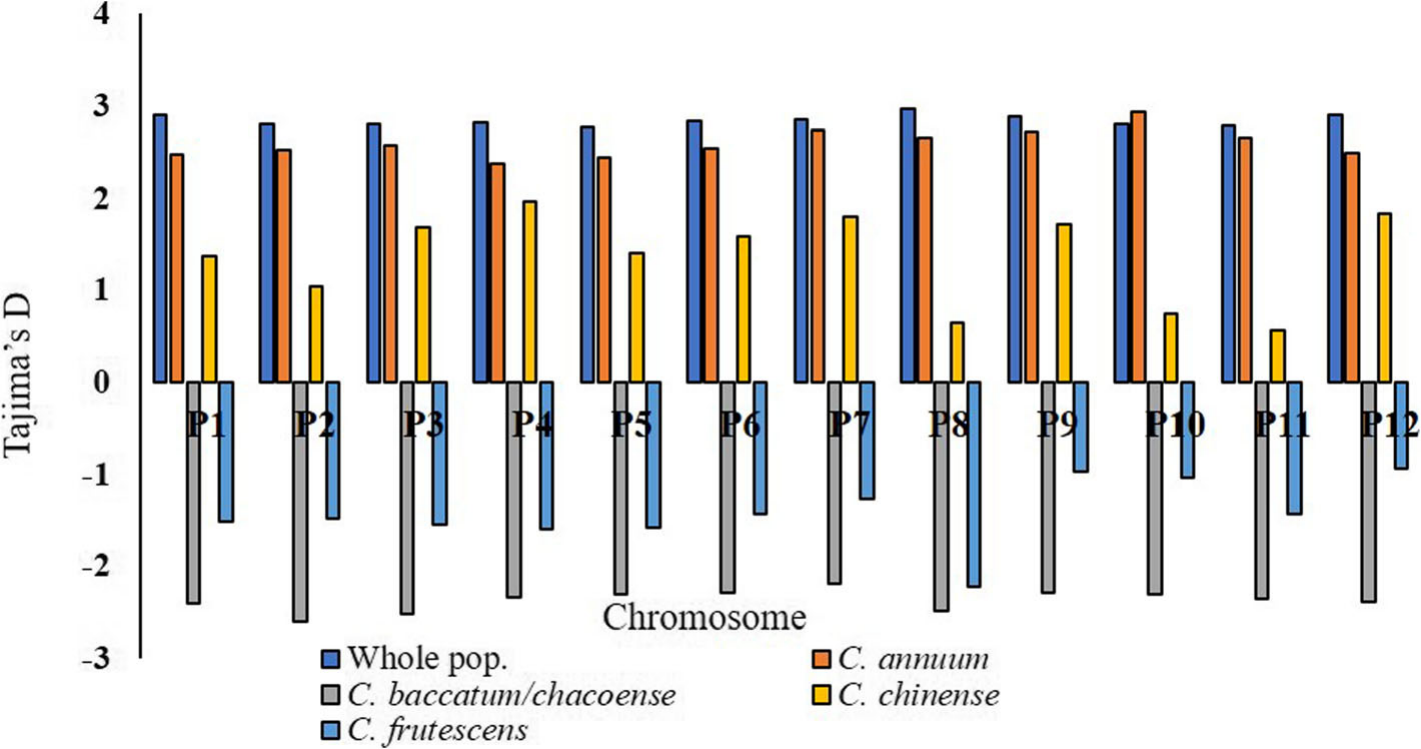
Tajima’s D statistics for each chromosome for the whole *Capsicum* population and representative species

**Fig. 4:**
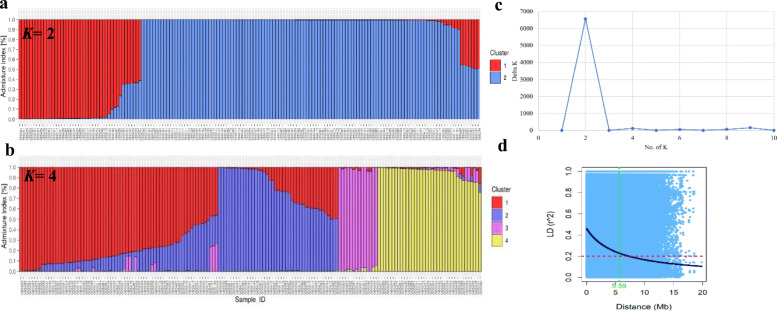
Bar plots for the admixture indices for each individual in the *Capsicum* population for *K*= 2 **a** and *K*= 4 **c** clusters. **b** Inference for the best number of clusters using the Evanno method revealed the optimal number of clusters to be *K*= 2. **d** Linkage disequilibrium (LD) decay plot for the *Capsicum* population. The red dashed line represents the critical value for LD (*r*^2^= 0.20) and the blue solid line represents the non-linear regression curve. The intersection between the critical value and the regression curve is the point at which LD starts to decay (~5.59 Mb)

Tajima’s D statistic for the *Capsicum* population across all chromosomes was D = 2.85 (Fig. 3). Within the individual chromosomes, P8 had the greatest value for D (2.97), followed by P1 and P12 (D = 2.91). Chromosome P5 had the lowest value for Tajima’s statistic (D = 2.78). Negative values for D were observed for the individual species. Within the clusters, Group II (*C. baccatum* and *C. chacoense*) with D= -2.39 had the least value for Tajima’s coefficient, followed by Group III (*C. frutescens*) with D= -1.41. Group I (*C. annuum* and *C. annuum* var. *glabriusculum*) had a D value of -0.19, whereas Group IV (*C. chinense*) had a value of -0.39. Chile pepper cultivars previously released by the NMSU Chile Pepper Breeding Program had a D value of -0.29.

**Table 2:** Genetic diversity indices for the *Capsicum* population

Pop	Species	Num^a^	Eff_Num	*H*_*o*_	*H*_*s*_	*G*_*is*_		π	θ	Tajima’s D		PIC	
I	*C. annuum*	1.94	1.21	0.04	0.13	0.70		0.12	0.13	-0.19		0.10	
II	*C. baccatum* & *C. chacoense*	1.23	1.07	0.04	0.06	0.34		0.06	0.09	-2.39		0.02	
III	*C. frutescens*	1.27	1.12	0.05	0.08	0.44		0.08	0.11	-1.41		0.05	
IV	*C. chinense*	1.90	1.31	0.10	0.20	0.51		0.17	0.19	-0.39		0.12	
Whole pop.		2.00	1.14	0.06	0.12	0.55		0.33	0.18	2.85		0.30	

^a^*Num*- Number of alleles; *Eff_Num * Effective number of alleles; *H*_*o *_Observed heterozygosity. *H*_*s *_Gene diversity; *G*_*is *_Inbreeding coefficient; *π* Observed nucleotide diversity; *θ* Expected nucleotide diversity. *PIC* Polymorphism information content

### Population structure and linkage disequilibrium

Inference for the best number of clusters, *K* using the Evanno criterion revealed *K* = 2 (*ΔK* = 6572.84) (Fig. 4a, b; Additional file 2, Table S2) to be the optimal number that best represents the *Capsicum* population. Cluster 1 comprised of *C. frutescens* and *C. chinense* (*N* = 44 genotypes), whereas cluster 2 consisted of the *C. annuum, C. baccatum*, and *C. chacoense* (*N* = 121) (Additional file 2, Table S3). In addition, *K* = 9 and *K* = 4 showed high *ΔK* relative to the other clusters, which indicates that these can also serve as alternative values to describe the genetic differentiation in the *Capsicum* population. For *K* = 4 (*ΔK* = 110.73; Fig. 4c), *C. annuum* genotypes were divided into two clusters, where cluster 1 was an admixed of 71 genotypes, including 22 chiltepins and 49 ornamental, chile piquin, de arbol, jalapeno, and serrano types (Additional file 2, Table S4). Cluster 2 comprised of 43 *C. annuum* cultivars which consisted of either the New Mexican or paprika types. *C. baccatum*, *C. frutescens*, and *C. chacoense* complexes were grouped in cluster 3, whereas cluster 4 consisted of the *C. chinense* genotypes.

Analysis of linkage disequilibrium (LD) identified more than 3.11 M intrachromosomal marker pairs across the 12 chromosomes of chile peppers (Additional file 2, Table S5). Mean values for LD coefficients (*r*^*2*^) ranged between 0.04 (P12) and 0.35 (P4). Average distance (in Mb) of all pairs was lowest for chromosomes P2 (0.59), P8 (0.70), and P3 (0.73). At least 80 % of the pairs were in significant LD (*P* < 0.05) across all chromosomes, with chromosome P1 having the largest percentage of significant marker pairs (84.40 %). Chromosome P2 had the least average distance of pairs in significant LD (0.61), followed by P8 and P3 (both with 0.77), and P6 (0.97). Total number of marker pairs in complete LD (*r*^*2*^ = 1.0) was 82,808 (2.65 %). Chromosome P3 had the highest number of pairs in complete LD (13,720), followed by P8 and P2, with 10,386, and 9,062 marker pairs, respectively. Chromosome P1 had only 23 intrachromosomal pairs in complete LD. The average distance of marker pairs in complete LD ranged between 0.40 (P1) and 2.12 Mb (P11). Analysis of LD decay by plotting *r*^*2*^ against distance revealed an extensive LD for the whole population, where LD starts to decay at ~ 5.59 Mb (Fig. 4d). Within the individual chromosomes, LD extends up to 14.78 Mb for chromosome P5. LD starts to decay at 0.07 and 0.38 Mb for the *C. annuum* and *C. chinense* complexes, respectively.

## Discussion

Evaluation of diversity is relevant for broadening the genetic base for identification of beneficial alleles for improvement of current germplasm [24]. A GBS approach was used for SNP marker discovery and to examine genetic diversity, population structure, and linkage disequilibrium among a diverse New Mexican *Capsicum* population. This panel included at least 50 different cultivars previously released by the NMSU Chile Pepper Breeding Program, regarded as the longest continuous program for *Capsicum* improvement in the world. Genomic information from this study would be useful for the genome wide selection and association studies for trait improvement in chile peppers.

### Genetic relatedness in New Mexican chile pepper germplasm

Majority of the SNP markers aligned to the *Zunla-1* reference genome (88 %), where only 12 % have unknown mapped positions. This number of SNP markers successfully aligned to the reference sequence was higher compared to that of Pereira-Dias et al. [21] and Taranto et al. [22] who observed 40.8 and 43.4 % of SNP markers mapped to CM-334, respectively. This could be a consequence of having mostly *C. annuum* genotypes in the population and the reference genome used. The presence of more transition substitutions on our population were consistent with other observations in chile peppers [21, 22, 24] supporting a ‘transition bias’ [28], which was related to the conservative effects of transitions on the corresponding protein products [29]. Moreover, we observed low levels of heterozygosity (5.30 %) in the *Capsicum* population that could be attributed to the inbreeding nature of the *Capsicum* spp. [22]. Genetic diversity for this *Capsicum* panel was relatively low, as indicated by various measures of diversity. Observed heterozygosity (*H*_*o*_) was relatively lower compared to Chinese and Spanish chile pepper populations previously evaluated by Du et al. [30] and González-Pérez et al. [31], respectively, but higher than that of an Ethiopian pepper germplasm assessed by Solomon et al. [24]. Gene diversity (*H*_*s*_) was also lower than that of a chile pepper population from China [32]. The relatively low genetic diversity on our *Capsicum* population indicates a need to broaden the current germplasm base for New Mexican chiles by introducing novel alleles from other pepper breeding program or through introgression of genes from the wild species.

Principal components analysis (PCA) revealed four distinct clusters based on species. *C. annuum* formed a cluster, whereas the other cultivated species, *C. baccatum*, *C. frutescens*, and *C. chinense* clustered into separate groups. Analysis of molecular variance further supported this differentiation, as majority of the variation (76.08 %) was attributed to the genetic differences among the populations. Previously, *C. annuum* was also observed to form a discrete group from other *Capsicum* species [21, 33]. Nonetheless, in contrast with the observations by Pereira-Dias et al. [21], we observed that the chiltepins clustered with the *C. annuum* in the PCA biplot. In the current study, the wild species *C. chacoense* grouped with *C. baccatum*, similar to earlier observations based on plastid DNA markers [34], a possible consequence of similar geographic origins for these species. *C. chacoense* also formed a cluster with *C. baccatum*, together with other wild *Capsicum* species evaluated in a large germplasm collection [35]. Another study, nevertheless, found *C. chacoense* accessions to be equally related to the *C. annuum*, *C. baccatum*, and *C. pubescens* complexes [36], whereas more recently, *C. chacoense* was placed between *C. baccatum* and *C. pubescens* [31]. Although close genetic relationships between *C. chinense* and C. *frutescens* have been shown using microsatellites and amplified fragment length polymorphism markers [37], we observed these species forming distinct clusters based on PCA. A relatively large marker dataset, such as the one used in the current study, might result in a more precise and robust clustering based on species in the PCA plot. The efficiency of utilizing a smaller subset of markers (i.e., 48 SNP loci) with high polymorphism content in combination with 32 different phenotypic traits, nevertheless, was previously demonstrated for the construction of a core collection of chile pepper germplasm [35]. Altogether, the varying patterns of clustering of the *Capsicum* spp. observed across different studies could result from the type of DNA-based marker, the representative genotypes evaluated, as well as the total number of loci used to differentiate the species.

Within the NMSU cultivars, the representative *C. chinense* genotypes formed a group with the *C. frutescens* (Fig. 2), indicating a close genetic relationship. The NMSU *C. annuum* complex separated into subgroups based on fruit type, consistent with previous observations among Spanish *C. annuum* pepper genotypes [31]. Breeding and selection for improvement of heirloom cultivars including ‘NuMex Big Jim’ and ‘NuMex Sandia’ have resulted in the release of the ‘NuMex Heritage Big Jim’ and the ‘NuMex Sandia Select’, with both cultivars having increased consumer and horticultural value [38, 39]. Genotyping using genome wide SNP markers showed that these improved heirloom cultivars did not necessarily cluster with the parental heirlooms, albeit still observed to be closely related cultivars. Neighbor-joining analysis based on SNP loci showed ‘NuMex Heritage Big Jim’ and ‘NuMex Sandia Select’ forming a group, whereas ‘NuMex Big Jim’ and ‘NuMex Sandia’ formed separate clusters with other New Mexican types. Such differences in alleles present at certain SNP sites between the parental and modern heirloom cultivars could be the result of multiple cycles of phenotypic recurrent selection combined with extensive single plant selections consequently leading to different SNP alleles present in the improved heirlooms.

### Selective sweeps in the chile pepper genome

The presence of potential selective sweeps in the chile pepper population and across the different *Capsicum* species was assessed using the Tajima’s D statistic. We observed a positive value for Tajima’s D statistic (D = 2.85) for the whole population, demonstrating an abundance of intermediate frequency alleles and can result from population structure, bottlenecks, and/or balancing selection [40]. This could also indicate that speciation or domestication for this *Capsicum* population have occurred at multiple sites, consequently contributing to genetic diversity [41]. Low (negative) values for the Tajima’s coefficient were nevertheless observed within the representative *Capsicum* species evaluated, demonstrating that minor alleles were less frequent than would be predicted in a neutrally evolving population, suggesting the potential occurrence of genes or gene clusters under strong purifying selection, population expansions, or positive selection [6, 40]. Contrarily, different *C. annuum* genotypes were recently observed to possess positive D values which could be possibly related with domestication and the accumulation of different trait-related mutations [21]. Consistent with our results, Pereira-Dias et al. [21] also reported lower Tajima’s D values for *C. chinense* and *C. frutescens* accessions, indicating the presence of rare alleles at low frequencies for these species. The varying values of Tajima’s D could signify that breeding and selection for the development of new cultivars across the different *Capsicum* species resulted in differences in the allele frequency. Positive D values were noted across individual chromosomes for the *C. annuum* complex, with chromosome P10 having the highest value for Tajima’s statistic (D = 2.93). The exposure to different breeding and selection processes, as well as the subsequent diversification of *C. annuum* cultivars might explain genetic diversity and recovery from genetic bottleneck effects in the individual chromosome level [41]. Altogether, differences in Tajima’s D values reflect the diverse processes that each New Mexican *Capsicum* species has undergone during breeding and selection.

### Population stratification in the ***Capsicum*** population

Analysis of population structure using a Bayesian model-based clustering algorithm revealed *K* = 2 as the optimal number of clusters for the *Capsicum* population. In this scenario, *C. chinense* and *C. frutescens* grouped together on a single cluster, further indicating a close genetic relationship between these species. Differences on the clustering of species between the PCA and the Bayesian approach were observed. These discrepancies could be a consequence of the differences on the process implemented on each analysis, and several factors including linkage disequilibrium (LD), number of markers, and the representative accessions used. Background LD, for example, can affect the accurate identification of population stratification in STRUCTURE, but not necessarily in PCA, where a high LD increases the probability of detecting spurious clustering among the individuals [42]. Results from PCA are also affected by the amount of data (markers) [43]. It should be noted that in interpreting the optimal *K*, the “biological significance” of the results should be highlighted, as the inferred *K* might not necessarily render biological relevance due to it being identified purely by a specified sampling scheme [44]. Therefore, in our case, *K* = 4 might give the most biological meaning among the inferred clusters, as this demonstrated clustering based on the number of representative *Capsicum* species evaluated. Clustering based on *K =* 4 further supported the differentiation based on fruit shapes or types, as the ornamental, piquin, and de arbol types, among others, were grouped on the same cluster, consistent with the current observations for a NJ analysis across the NMSU chile pepper cultivars (Fig. 2).

### Extensive linkage disequilibrium

Knowledge of the LD decay across populations is relevant for the identification of significant marker-trait associations and implementation of marker-assisted selection [45, 46]. In the current study, analyses revealed varying levels of LD decay across the different chromosomes for the *Capsicum* population. Extensive LD was observed for the whole population, with LD reaching to ~ 5.59 Mb, whereas a rapid LD decay was noted for the *C. annuum* (0.07 Mb) and *C. chinense* (0.38 Mb) complexes for the evaluated *Capsicum* population. Our results for the *C. annuum* was consistent with Taranto et al. [22] who also observed a rapid decay of LD at 0.01 Mb. The extensive LD decay across the whole *Capsicum* indicates that a lower number of markers would be required in implementing genome wide association studies for identifying significant marker-trait associations. Conversely, for the *C. annuum* and *C. chinense* complexes, a higher number of markers would be needed for association mapping as a consequence of a rapid decay of LD.

## Conclusions

Information on genetic diversity is relevant for the genomic improvement of current germplasm. The present study provided insights into the genetic diversity of 165 *Capsicum* cultivars using GBS-derived SNP markers. Analysis of principal components revealed distinct groups based on species. *C. annuum*, *C. frutescens*, and *C. chinense* formed distinct clusters, whereas *C. baccatum* and *C. chacoense* clustered together in a group. A Bayesian clustering approach showed the optimal number of cluster *K* to be equal to 2. The NMSU chile pepper cultivars clustered according to species, where the *C. annuum* grouped together based on fruit or pod type, and the *C. chinense* and *C. frutescens* grouped in a single cluster. Presence of positive selection, population bottleneck, and balancing selection has been observed in the *Capsicum* population. The extensive LD observed for the *Capsicum* panel indicates that a lower number of markers can be used for genome wide association mapping. The relatively low genetic diversity in the current New Mexican *Capsicum* population could be improved by introducing novel alleles from other breeding programs or from wild germplasm. We present valuable information for future genome wide selection and genetic mapping studies for different horticultural traits in New Mexican chile peppers.

## Methods

### Plant material

A collection of *Capsicum* lines consisting of 165 diverse genotypes of chile peppers from five *Capsicum* species was evaluated in the current study (Additional file 1, Table S6)**.** In total, 91 genotypes belong to *C. annuum*, whereas 23 lines are classified as *C. annuum* var. *glabriusculum* (chiltepins). There were six lines belonging to *C. baccatum*, 37 *C. chinense*, and seven genotypes for *C. frutescens*. In addition to the cultivated chile peppers, one accession from the wild *Capsicum* species, *C. chacoense*, was included in the study.

Among those belonging to the *Capsicum* population were 53 NMSU chile pepper cultivars from three different cultivated species, *C. annuum*, *C. chinense*, and *C. frutescens*. These cultivars possess different fruit (pod) types such as New Mexican, paprika, cayenne, jalapeno, and included the ornamental chile peppers specifically developed for the potted plant and nursery industries [47], all belonging to *C. annuum*. The New Mexican types included ‘NuMex Joe E. Parker’ [48], ‘NuMex Heritage Big Jim’ [38], ‘NuMex Big Jim’ [49], and ‘NuMex Sandia Select’ [39], whereas the cayenne type included ‘NuMex Las Cruces’ [50]. The paprika type consisted of ‘NuMex Garnet’ [51], and the jalapenos comprised of ‘NuMex Jalmundo’ [52], ‘NuMex Vaquero’ [53], and ‘NuMex Piñata’ [54]. The ornamental types included ‘NuMex Twilight’ [55], ‘NuMex Christmas’, and ‘NuMex Thanksgiving’ [56]. The *C. chinense* comprised of the ‘NuMex Trick- or- Treat’ [57], a no-heat habanero, and ‘NuMex Suave Red’ and ‘NuMex Suave Orange’ [58], whereas *C. frutescens* consisted of the ‘NuMex Nobasco’ [59], a no-heat type tabasco. The ajis (*C. baccatum*) included the “Aji Guyana’ from the Andrean region of South America. Finally, the *Capsicum* population also included some of the ‘Superhot’ chile peppers (*C. chinense*) with average Scoville heat levels reaching to at least 1 million, such as the ‘Trinidad Scorpion’ and ‘Carolina Reaper’ (https://puckerbuttpeppercompany.com), regarded as the hottest chile pepper in the world.

### DNA extraction and quantification

Seeds were planted in F1020 insert multi-cell trays (American Horticultural Supply, Inc., CA, USA) at the Fabian Garcia Research Center, NMSU, Las Cruces, NM and were grown and maintained under standard greenhouse conditions. Leaf tissues of 30-45-day old chile pepper seedlings from single plants were collected using 1.2 mL Qiagen® polypropylene collection microtubes (Qiagen, MD, USA). Approximately 50 mg of fresh leaf tissue samples were used for extraction using Qiagen DNeasy® 96 plant extraction kits following manufacturer’s protocol through the University of Minnesota Genomics Center DNA Extraction facility (https://genomics.umn.edu/service/dna-extraction). Quantification of DNA was done using Picogreen® (Thermofisher Scientific, MA, USA) and samples were normalized to 10 ng/µl for sequencing.

### Genotyping-by-sequencing library preparation and genotyping

Genotyping-by-sequencing (GBS) for the *Capsicum* population was conducted through the University of Minnesota Genomics Center (https://genomics.umn.edu/services/gbs) using a single enzyme digestion protocol. Briefly, extracted DNA was quantified using Picogreen® (Thermofisher Scientific, MA, USA) and normalized to 10 ng/µl. A total of 100 ng of DNA per sample was digested with 10 units of *ApeKI* (New England Biolabs®, Inc. MA, USA) restriction enzyme and incubated at 75^0^ C for 2 h. The DNA samples were then ligated with 200 units of T4 ligase (New England Biolabs®, Inc. MA, USA) and phased adaptors with -CWG overhang at 22^0^ C for 1 h and heat killed. The ligated samples were then purified with solid phase reversible immobilization (SPRI) beads and then amplified for 18 cycles with 2X NEB Taq Master Mix to add the barcodes. Libraries were SPRI purified, quantified, and pooled. Fragments with the 300–744 bp size region were selected and diluted to 1 nM for sequencing on the Illumina NovaSeq™ 6000 (Illumina, CA, USA) using single end 1 × 100 reads.

The raw FASTQ files were demultiplexed using the Illumina bcl2fastq software (Illumina, CA, USA). The first 12 bases were removed from the beginning of each read in order to remove adapter sequences. Trimmomatic [60] was used to remove adapter sequences at the 3’ ends of the reads. The FASTQ files were aligned to the *Zunla-1* (*C. annuum*) reference genome [8] using the Burrows-Wheeler Aligner [61]. Freebayes [62] was used to jointly call variants across all samples simultaneously. The raw variant call format (VCF) files were processed using VCFtools to remove variants with minor allele frequency < 1 %, genotype rates < 95 %, and samples with genotype rates < 50 %. The VCF files were converted to HapMap format using TASSEL 5.2.67 [63, 64], where SNP markers with MAF < 0.05 and minor states were further excluded. Imputation of missing data was conducted using the LD *k*-nearest neighbor genotype imputation function [65] in TASSEL 5.2.67 [63, 64]. HapMap was transformed to numeric data format using the iPAT program [66].

### Analysis of principal components, molecular variance, and genetic diversity

Principal components analysis using genome-wide SNP markers were conducted using the “PCA for Population Stratification” function in JMP Genomics [67]. Analysis of molecular variance (AMOVA) [68] was implemented using the ‘poppr’ package [69] in the statistical package R [70]. Observed nucleotide diversity or average pairwise divergence (π), estimated mutation rate or expected nucleotide diversity (θ) [71], and Tajima’s D statistic [72], used to assess the presence of selective sweeps in New Mexican chile peppers, were calculated using TASSEL 5.2.67. Various measures of genetic diversity including observed heterozygosity (*H*_*o*_), heterozygosity within populations (*H*_*s*_), total heterozygosity (*H*_*t*_), and inbreeding coefficient (*G*_*is*_) were calculated for the *Capsicum* population using the GenoDive program [73]. Fixation index (*F*_*st*_) among the different *Capsicum* species was calculated using the ‘Population Measures’ function in JMP Genomics. Polymorphism information content (PIC) was computed using the ‘GeneticSubsetter’ package [74] in R.

### Population structure and linkage disequilibrium

Genetic stratification for the chile pepper population was evaluated using the program STRUCTURE [44]. An admixture model was applied with the following criteria: burn-in of 10,000 iterations, 10,000 Monte Carlo Markov Chain replicates, and number of clusters *K* between 1 and 10, with the number of replications per *K* equal to 5. Inference on the true number of *K* that best represent the genotypes was conducted with the Evanno criterion that employs an ad hoc statistic *ΔK* based on the degree of changes in the log probability of data between values of *K* [75] implemented in STRUCTURE HARVESTER [76]. Admixture indices derived from STRUCTURE for each sample were visualized through bar plots using the ‘StructuRly’ program [77]. Linkage disequilibrium (LD) analysis for intrachromosomal marker pairs was conducted in TASSEL 5.2.67. Coefficients of LD were represented as the square of allele frequency correlations between pairs of loci, *r*^*2*^ [78]. The pairwise *r*^*2*^ values were plotted against genetic physical distance (in Mb) and a non-linear regression model [79, 80] was fitted to the LD plot. The intersection between the critical value (*r*^*2*^ = 0.20; [22]) and the regression curve was regarded as the distance at which LD starts to decay. Intrachromosomal marker pairs with *P* < 0.05 were declared to be in significant LD.

## Supplementary Information

**Additional file 1:** **Table S1.** Flanking sequences for the 66,750 SNP markers identified in the *Capsicum* population.

**Additional file 2:** **Table S1.** Fixation indices (*F*_*st*_) across the different Capsicum species. **Table S2.** Inference on the best number of clusters *K* for the *Capsicum* population using Evanno criterion. **Table S3.** Distribution of the *Capsicum* genotypes based on hierarchical and Bayesian-based model clustering for number of clusters, *K* = 2. Inferred group designation for each entry is highlighted. **Table S4.** Distribution of the *Capsicum* genotypes based on hierarchical and Bayesian-based model clustering for number of clusters, *K*= 4. Inferred group designation for each entry is highlighted. **Table S5.** Overview of linkage disequilibrium for the intrachromosomal marker pairs in the *Capsicum* population. **Table S6.** Details of the 165 *Capsicum* genotypes used in this study.

## Data Availability

The datasets generated and/or analyzed during the current study are available in the FigShare repository, www.10.6084/m9.figshare.14447526 (Accession number: 14,447,526) and www.10.6084/m9.figshare.14447733 (Accession number: 14,447,733).

## Abbreviations

GBS: Genotyping-by-sequencing
LD: Linkage disequilibrium
PCA: Principal components analysis
SNP: Single nucleotide polymorphisms
